# Dietary Strategies Implicated in the Prevention and Treatment of Metabolic Syndrome

**DOI:** 10.3390/ijms17111877

**Published:** 2016-11-10

**Authors:** Rocio de la Iglesia, Viviana Loria-Kohen, Maria Angeles Zulet, Jose Alfredo Martinez, Guillermo Reglero, Ana Ramirez de Molina

**Affiliations:** 1 GENYAL Platform on Nutrition and Health, IMDEA Food Institute, CEI UAM + CSIC, 28049 Madrid, Spain; viviana.loria@imdea.org (V.L.-K.); guillermo.reglero@imdea.org (G.R.); 2 Department of Nutrition, Food Science and Physiology, University of Navarra, 31008 Pamplona, Spain; mazulet@unav.es (M.A.Z.); jalfmtz@unav.es (J.A.M.); 3 CIBER Fisiopatología de la Obesidad y la Nutrición (CIBERobn), 28029 Madrid, Spain

**Keywords:** metabolic syndrome, dietary strategies, bioactive compounds

## Abstract

Metabolic syndrome (MetS) is established as the combination of central obesity and different metabolic disturbances, such as insulin resistance, hypertension and dyslipidemia. This cluster of factors affects approximately 10%–50% of adults worldwide and the prevalence has been increasing in epidemic proportions over the last years. Thus, dietary strategies to treat this heterogenic disease are under continuous study. In this sense, diets based on negative-energy-balance, the Mediterranean dietary pattern, n-3 fatty acids, total antioxidant capacity and meal frequency have been suggested as effective approaches to treat MetS. Furthermore, the type and percentage of carbohydrates, the glycemic index or glycemic load, and dietary fiber content are some of the most relevant aspects related to insulin resistance and impaired glucose tolerance, which are important co-morbidities of MetS. Finally, new studies focused on the molecular action of specific nutritional bioactive compounds with positive effects on the MetS are currently an objective of scientific research worldwide. The present review summarizes some of the most relevant dietary approaches and bioactive compounds employed in the treatment of the MetS to date.

## 1. The Metabolic Syndrome

It was during the period between 1910 and 1920 when it was suggested for the first time that a cluster of associated metabolic disturbances tended to coexist together [1]. Since then, different health organisms have suggested diverse definitions for metabolic syndrome (MetS) but there has not yet been a well-established consensus. The most common definitions are summarized in Table 1. What is clear for all of these is that the MetS is a clinical entity of substantial heterogeneity, commonly represented by the combination of obesity (especially abdominal obesity), hyperglycemia, dyslipidemia and/or hypertension [2,3,4,5,6].

Obesity consists of an abnormal or excessive fat accumulation, for which the main cause is a chronic imbalance between energy intake and energy expenditure [7,8]. The excess of energy consumed is primarily deposited in the adipose tissue as triglycerides (TG) [9].

Dyslipidemia encompasses elevated serum TG levels, increased low density lipoprotein-cholesterol (LDL-c) particles, and reduced levels of high density lipoprotein-cholesterol (HDL-c) [10]. It is associated with hepatic steatosis [11], dysfunction of pancreatic β-cells [12] and elevated risk of atherosclerosis [13], among others.

Another main modifiable MetS manifestation is hypertension, which is mainly defined as a resting systolic blood pressure (SBP) ≥ 140 mmHg or diastolic blood pressure (DBP) ≥ 90 mmHg or drug prescription to lower hypertension [14]. It usually involves narrowed arteries and is identified as a major cardiovascular and renal risk factor, related to heart and vascular disease, stroke and myocardial infarction [13,15,16,17].

Hyperglycemia, related insulin resistance and type 2 diabetes mellitus are characterized by an impaired uptake of glucose by the cells, that lead to elevated plasma glucose levels, glycosuria and ketoacidosis [18]. It is responsible for different tissue damage that shortens the life expectancy of diabetics, involving cardiovascular diseases (CVD), atherosclerosis, hypertension [19], β-cell dysfunction [12], kidney disease [20] or blindness [21]. Currently, diabetes is considered the leading cause of death in developed countries [22].

Moreover, oxidative stress and low grade inflammation are two important mechanisms implicated in the etiology, pathogenesis, and development of MetS [23]. Oxidative stress is defined as an imbalance between the pro-oxidants and antioxidants in the body [24]. It plays a key role in the development of atherosclerosis by different mechanisms such as the oxidation of LDL-c particles [25] or impairment of HDL-c functions [26]. Inflammation is an immune system response to injury hypothesized to be a major mechanism in the pathogenesis and progression of obesity related disorders and the link between adiposity, insulin resistance, MetS and CVD [27].

Although the prevalence of the MetS varies broadly around the word and depends on the source used for its definition, it is clear that over the last 40–50 years the number of people presenting with this syndrome has risen in epidemic proportions [28]. Moreover, the frequency of this syndrome is increased in developed countries, sedentary people, smokers, low socioeconomic status population, as well as in individuals with unhealthy dietary habits [29,30].

For all of this, there is currently a wide concern to find effective strategies to detect, treat and control the comorbidities associated with MetS. This is a complex challenge as MetS is a clinical entity of substantial heterogeneity and therefore, the different cornerstones implicated in its development should be addressed. In this review we compiled and examined different dietary patterns and bioactive compounds that have pointed out to be effective in MetS treatment.

## 2. Dietary Patterns

Several dietary strategies and their potential positive effects on the prevention and treatment of the different metabolic complications associated to the MetS, are described below and summarized in Table 2.

### 2.1. Energy-Restricted Diets

Energy restricted diets are probably the most commonly used and studied dietary strategies for combating excess weight and related comorbidities. They consist in personalized regimes that supply less calories than the total energy expended by a specific individual [31].

A hypocaloric diet results in a negative energy balance and subsequently, in body weight reduction [31]. Weight loss is achieved via fat mobilization from different body compartments as a consequence of the lipolysis process necessary to provide energy substrate [32,33]. In people who are overweight or suffering from obesity, as is the case of most people with MetS, weight loss is important as it is associated with improvement of related disorders such as abdominal obesity (visceral adipose tissue), type 2 diabetes, CVD or inflammation [32,33,34,35,36].

Moreover, as described above, low grade inflammation is associated with MetS and obesity. Therefore, of particular importance is the fact that in obese individuals following a hypocaloric diet, a depletion of plasma inflammatory markers such as interleukin (IL)-6 has been observed [34]. Thus, caloric restriction in obese people suffering MetS may improve the whole-body pro-inflammatory state.

At the same time, body weight reduction is associated with improvements in cellular insulin signal transduction, increments in peripheral insulin sensitivity and higher robustness in insulin secretory responses [32,36]. People with excess body weight who are at risk of developing type 2 diabetes, may benefit from a hypocaloric regime by improving plasma glucose levels and insulin resistance.

In addition, different intervention trials have reported a relationship between energy restricted diets and lower risk of developing CVD. In this sense, in studies with obese people following a hypocaloric diet, improvements in lipid profile variables such as reductions of LDL-c and plasma TG levels, as well as improvements in hypertension via depletion of SBP and DBP levels have been observed [35,37].

Among the different nutritional intervention trials, a reduction of 500–600 kcal a day of the energy requirements is a well-established hypocaloric dietary strategy, which has demonstrated to be effective in weight reduction [38,39]. However, the challenge lies in maintaining the weight loss over time, as many subjects can follow a prescribed diet for a few months, but most people have difficulty in maintaining the acquired habits over the long term [40,41].

### 2.2. Diets Rich in Omega-3 Fatty Acids

The very long-chain eicosapentaenoic acid (EPA) and docosahexaenoic acid (DHA) are essential omega-3 polyunsaturated fatty acids (n-3 PUFAs) for human physiology. Their main dietary sources are fish and algal oils and fatty fish, but they can also be synthesized by humans from α-linolenic acid [40].

There is a moderate body of evidence suggesting that n-3 PUFAs, mainly EPA and DHA, have a positive role in the prevention and treatment of the pathologies associated to MetS [42].

In this context, it has been described that EPA and DHA have the ability to reduce the risk of developing CVD and cardiometabolic abnormalities as well as CVD-related mortality [42]. These beneficial effects are thought to be mainly due to the ability of these essential fatty acids to reduce plasma TG levels [43].

Moreover, different studies have shown that people following an increased n-3 PUFA diet have reduced plasma levels of the pro-inflammatory cytokines IL-6 and tumor necrosis factor-alpha (TNFα), as well as plasma C-reactive protein (CRP) [44]. These effects are probably mediated by resolvins, maresins and protectins, which are EPA and DHA metabolic products with anti-inflammatory properties [44].

There are some studies that have observed an association between n-3 ingestion and improvements or prevention of type 2 diabetes development. However, other studies found opposite results [44]. Thus, it cannot be made any specific affirmation in this respect.

The European Food Safety Authority recommends and intake of 250 mg EPA + DHA a day, in the general healthy population as a primary prevention of CVD [45]. These amounts can be achieved with an ingestion of 1–2 fatty fish meals per week [45].

### 2.3. Diets Based on Low Glycemic Index/Load

Over the last ten years, the concern about the quality of the carbohydrates (CHO) consumed has risen [46]. In this context, the glycemic index (GI) is used as a CHO quality measure. It consists in a ranking on a scale from 0 to 100 that classifies carbohydrate-containing foods according to the postprandial glucose response [47]. The higher the index, the more promptly the postprandial serum glucose rises and the more rapid the insulin response. A quick insulin response leads to rapid hypoglycemia, which is suggested to be associated with an increment of the feeling of hunger and to a subsequent higher caloric intake [47]. The glycemic load (GL) is equal to the GI multiplied by the number of grams of CHO in a serving [48].

There is a theory which states that MetS is a consequence of an elevated intake of high GI foods over time, among others unhealthy dietary habits [49]. In this sense, following a diet rich in high GI CHO has been associated with hyperglycemia, insulin resistance, type 2 diabetes, hypertriglyceridemia, CVD, and obesity [47,50,51], abnormalities directly related to MetS.

On the contrary, a low GI diet has been associated with slower absorption of the CHO and subsequently smaller blood glucose fluctuations, which indicate better glycemic control [46]. In patients with type 2 diabetes, diets based on low GI are associated with reductions in glycated hemoglobin (HbA1c) and fructosamine blood levels, two biomarkers used as key monitoring factors in diabetes management [52,53].

For all of this, it is common to find the limitation of CHO at high GI among the advice for MetS treatment [28], in particular with respect to “ready-to-eat processed foods” including sweetened beverages, soft drinks, cookies, cakes, candy, juice drinks, and other foods which contain high amounts of added sugars [54].

### 2.4. Diets with High Total Antioxidant Capacity

Dietary total antioxidant capacity (TAC) is an indicator of diet quality defined as the sum of antioxidant activities of the pool of antioxidants present in a food [55]. These antioxidants have the capacity to act as scavengers of free radicals and other reactive species produced in the organisms [56]. Taking into account that oxidative stress is one of the remarkable unfortunate physiological states of MetS, dietary antioxidants are of main interest in the prevention and treatment of this multifactorial disorder [57]. Accordingly, it is well-accepted that diets with a high content of spices, herbs, fruits, vegetables, nuts and chocolate, are associated with a decreased risk of oxidative stress-related diseases development [58,59,60]. Moreover, several studies have analyzed the effects of dietary TAC in individuals suffering from MetS or related diseases [61,62]. In the Tehran Lipid and Glucose Study it was demonstrated that a high TAC has beneficial effects on metabolic disorders and especially prevents weight and abdominal fat gain [61]. In the same line, research conducted in our institutions also evidenced that beneficial effects on body weight, oxidative stress biomarkers and other MetS features were positively related with higher TAC consumption in patients suffering from MetS [63,64,65].

In this sense, the World Health Organization (WHO) recommendation for fruit and vegetables consumption (high TAC foods) for the general population is a minimum of 400 g a day [66]. Moreover, cooking with spices is recommended in order to increase the TAC dietary intake and, at the same time, maintain flavor while reducing salt [67].

### 2.5. Moderate-High Protein Diets

The macronutrient distribution set in a weight loss dietary plan has commonly been 50%–55% total caloric value from CHO, 15% from proteins and 30% from lipids [57,68]. However, as most people have difficulty in maintaining weight loss achievements over time [69,70], research on increment of protein intake (>20%) at the expense of CHO was carried out [71,72,73,74,75,76,77].

Two mechanisms have been proposed to explain the potential beneficial effects of high-moderate protein diets: the increment of diet-induced thermogenesis [73] and the increase of satiety [78]. The increment of the thermogenesis is explained by the synthesis of peptide bonds, production of urea and gluconeogenesis, which are processes with a higher energy requirement than the metabolism of lipids or CHO [75]. An increment of different appetite-control hormones such as insulin, cholecystokinin or glucagon-like peptide 1, may clarify the satiety effect [79].

Other beneficial effects attributed to moderate-high protein diets in the literature are the improvement of glucose homeostasis [80], the possibility of lower blood lipids [81], the reduction of blood pressure [82], the preservation of lean body mass [83] or the lower of cardiometabolic disease risk [84,85]. However, there are other studies that have not found benefits associated to a moderate-high protein diet [76]. This fact may be explained by the different type of proteins and their amino acid composition [80], as well as by the different type of populations included in each study [85]. Therefore, more research in the field is needed in order to make these results consistent.

In any case, when a hypocaloric diet is implemented, it is necessary to slightly increase the amount of proteins. Otherwise it would be difficult to reach the protein energy requirements, established as 0.83 g/kg/day for isocaloric diets and which should probably be at least 1 g/kg/day for energy-restricted diets [86].

### 2.6. High Meal Frequency Pattern

The pattern of increasing meal frequency in weight loss and weight control interventions has currently become popular among professionals [87,88]. The idea is to distribute the total daily energy intake into more frequently and smaller meals. However, there is no strong evidence about the efficacy of this habit yet [89]. While some investigations have found an inverse association between the increment of meals per day and body weight, body mass index (BMI), fat mass percentage or metabolic diseases such as coronary heart disease or type 2 diabetes [71,88,90,91,92], others have found no association [93,94,95].

Different mechanisms by which high meal frequency can have a positive effect on weight and metabolism management have been proposed. An increment of energy expenditure was hypothesized; however, the studies carried out in this line have concluded that total energy expenditure does not differ among different meal frequencies [96,97]. Another postulated hypothesis is that the greater the number of meals a day, the higher the fat oxidation, but again no consensus has been achieved [89,98]. An additional suggested mechanism is that increasing meal frequency leads to plasma glucose levels with lower oscillations and reduced insulin secretion which is thought to contribute to a better appetite control. However, these associations have been found in population with overweight or high glucose levels but in normal-weight or normoglycaemic individuals the results are still inconsistent [93,99,100,101].

### 2.7. The Mediterranean Diet

The concept of the Mediterranean Diet (MedDiet) was for the first time defined by the scientific Ancel Keys who observed that those countries around the Mediterranean Sea, which had a characteristic diet, had less risk of suffering coronary heart diseases [102,103].

The traditional MedDiet is characterized by a high intake of extra-virgin olive oil and plant foods (fruits, vegetables, cereals, whole grains, legumes, tree nuts, seeds and olives), low intakes of sweets and red meat and moderate consumption of dairy products, fish and red wine [104].

There is a lot of literature supporting the general health benefits of the MedDiet. In this sense, it has been reported that a high adherence to this dietary pattern protects against mortality and morbidity from several causes [105]. Thus, different studies suggested the MedDiet as a successful tool for the prevention and treatment of MetS and related comorbidities [106,107,108]. Moreover, recent meta-analysis concluded that the MedDiet is associated with less risk of developing type 2 diabetes and with a better glycemic control in people with this metabolic disorder [107,109,110]. Other studies have found a positive correlation between the adherence to a MedDiet pattern and reduced risk of developing CVD [111,112,113,114]. In fact, many studies have found a positive association between following a MedDiet and improvements in lipid profile by reduction of total cholesterol, LDL-c and TG, and an increase in HDL-c [111,112,113,114,115]. Finally, different studies also suggest that the MedDiet pattern may be a good strategy for obesity treatment as it has been associated with significant reductions in body weight and waist circumference [108,116,117].

The high amount of fiber which, among other beneficial effects, helps to weight control providing satiety; and the high antioxidants and anti-inflammatory nutrients such as n-3 fatty acids, oleic acid or phenolic compounds, are thought to be the main contributors to the positive effects attributed to the MedDiet [118].

For all these reasons, efforts to maintain the MedDiet pattern in Mediterranean countries and to implement this dietary habits in westernized countries with unhealthy nutritional patterns should be made.

## 3. Single Nutrients and Bioactive Compounds

New studies focused on the molecular action of nutritional bioactive compounds with positive effects on MetS are currently an objective of scientific research worldwide with the aim of designing more personalized strategies in the framework of molecular nutrition. Among them, flavonoids and antioxidant vitamins are some of the most studied compounds with different potential benefits such as antioxidant, vasodilatory, anti-atherogenic, antithrombotic, and anti-inflammatory effects [119]. Table 3 summarizes different nutritional bioactive compounds with potential positive effects on MetS, including the possible molecular mechanism of action involved.

### 3.1. Ascorbate

Vitamin C, ascorbic acid or ascorbate is an essential nutrient as human beings cannot synthesize it. It is a water-soluble antioxidant mainly found in fruits, especially citrus (lemon, orange), and vegetables (pepper, kale) [120]. Several beneficial effects have been associated to this vitamin such as antioxidant and anti-inflammatory properties and prevention or treatment of CVD and type 2 diabetes [121,122,123].

This dietary component produces its antioxidant effect primarily by quenching damaging free radicals and other reactive oxygen and nitrogen species and therefore preventing molecules such as LDL-c from oxidation [122]. It can also regenerate other oxidized antioxidants like tocopherol [124].

Moreover, it has been described that ascorbic acid may reduce inflammation as it is associated with depletion of CRP levels [125]. This is an important outcome to take in consideration in the treatment of MetS sufferers, as they usually present low grade inflammation [27].

Supplementation with vitamin C have also been associated with prevention of CVD by improving the endothelial function [126] and probably by lowering blood pressure [121]. These effects are thought to be exerted by the ability of vitamin C to enhance the endothelial nitric oxide synthase enzyme (eNOS) activity and to reduce HDL-c glycation [127].

Additionally, several studies have attributed to ascorbate supplementation an antidiabetic effect by improving whole body insulin sensitivity and glucose control in people with type 2 diabetes [123]. These antidiabetic properties are thought to be mediated by optimization of the insulin secretory function of the pancreatic islet cells by increasing muscle sodium-dependent vitamin C transporters (SVCTs) [128].

Despite all of this, it should be taken into account that most people reach ascorbic acid requirements (established at 95–110 mg/day in the general population) from diet and do not need supplementation [122,129]. Besides, it should be considered that an excess of vitamin C ingestion leads to the opposite effect and oxidative particles are formed [130,131].

### 3.2. Hydroxytyrosol

Hydroxytyrosol (3,4-dihydroxyphenylethanol) is a phenolic compound mainly found in olives [132].

It is considered the strongest antioxidant of olive oil and one of the main antioxidants in nature [133]. It acts as a powerful scavenger of free radicals, as a radical chain breaker and as metal chelator [134]. It has the ability of inhibiting LDL-c oxidation by macrophages [132]. In this sense, it is the only phenol recognized by the European Food Safety Authority (EFSA) as a protector of blood lipids from oxidative damage [135].

Hydroxytyrosol has also been reported to have anti-inflammatory effects, probably by suppressing cyclooxygenase activity and inducing eNOS expression [136]. Thus, enhancement of olives/olive oil intakes or hydroxytyroxol supplementation in people suffering from MetS may be a good strategy in order to improve inflammatory status.

Another beneficial effect attributed to this phenolic compound is its cardiovascular protective effect. It presents anti-atherogenic properties by decreasing the expression of vascular cell adhesion protein 1 (VCAM-1) and intercellular adhesion molecule 1 (ICAM-1) [132,137], which are probably the result of an inactivation of the nuclear factor kappa-light-chain-enhancer of activated B cells (NFκB), activator protein 1 (AP-1), GATA transcription factor and nicotinamide adenine dinucleotide phosphate (NAD(P)H) oxidase [138,139]. Hydroxytyrosol also provides antidyslipidemic effects by lowering plasma levels of LDL-c, total cholesterol and TG, and by rising HDL-c [138].

Despite the beneficial effects attributed to hydfroxytyrosol as an antioxidant, for its antiinflamatory properties and as cardiovascular protector, it should be taken into account that most studies focused on this compound have been performed with mixtures of olive phenols, thus a synergic effect cannot be excluded [140].

### 3.3. Quercetin

Quercetin is a predominant flavanol naturally present in vegetables, fruits, green tea or red wine. It is commonly found as glycoside forms, where rutin is the most common and important structure found in nature [141].

Many beneficial effects that can contribute to MetS improvement have been attributed to quercetin. Among them, its antioxidant capacity should be highlighted, as it has been reported to inhibit lipid peroxidation and increase antioxidant enzymes such as superoxide dismutase (SOD), catalase (CAT) or glutathione peroxidase (GPX) [142].

Moreover, an anti-inflammatory effect mediated via attenuation of tumor necrosis factor α (TNF-α), NFκB and mitogen-activated protein kinases (MAPK), as well as depletion of IL-6, IL-1β, IL-8 or monocyte chemoattractant protein-1 (MCP-1) gene expression has also been attributed to this polyphenol [143].

As most people with MetS are overweight or obese, the role of quercetin in body weight reduction and obesity prevention has been of special interest. In this sense, it stands out the capacity of quercetin to inhibit adipogenesis through inducing the activation of AMP-activated protein kinase (AMPK) and decreasing the expression of CCAAT-enhancer-binding protein-α (C/EBPα), peroxisome proliferator-activated receptor gamma (PPARγ), and sterol regulatory element-binding protein 1 (SREBP-1) [141,144].

According to the antidiabetic effects, it is proposed that quercetin may act as an agonist of peroxisome proliferator-activated receptor gamma (PPARγ), and thus improve insulin-stimulated glucose uptake in mature adipocytes [145]. Moreover, quercetin may ameliorate hyperglycemia by inhibiting glucose transporter 2 (GLUT2) and insulin dependent phosphatidylinositol-3-kinase (PI3K) and blocking tyrosine kinase (TK) [142].

Finally, different studies observed that quercetin has the ability to reduce blood pressure [146,147,148]. However, the mechanisms of action are not clear, since some authors have suggested that quercetin increases eNOS, contributing to inhibition of platelet aggregation and improvement of the endothelial function [146,147], but there are other studies that have not come across these results [148].

### 3.4. Resveratrol

Resveratrol (3,5,4′-trihidroxiestilben) is a phenolic compound mainly found in red grapes and derived products (red wine, grape juice) [149]. It has shown antioxidant and anti-inflammatory activities, and cardioprotective, anti-obesity and antidiabetic capacities [150,151,152,153,154,155,156].

The antioxidant effects of resveratrol have been reported to be carried out by scavenging of hydroxyl, superoxide, and metal-induced radicals as well as by antioxidant effects in cells producing reactive oxygen species (ROS) [150].

Moreover, it has been reported that the anti-inflammatory effects of resveratrol are mediated by inhibiting NFκB signaling [151]. Furthermore, this polyphenol reduces the expression of proinflammatory cytokines such as interleukin 6 (IL-6), interleukin 8 (IL-8), TNF-α, monocyte chemoattractant protein-1 (MCP-1) and eNOS [152]. In addition, resveratrol inhibits the cyclooxygenase (COX) expression and activity, a pathway involved in the synthesis of proinflammatory lipid mediators [152].

Concerning the effects of resveratrol against development of type 2 diabetes, it has been reported that treatment of diabetes patients with this polyphenol provides significant improvements in the status of multiple clinically relevant biomarkers such as fasting glucose levels, insulin concentrations or glycated hemoglobin and Homeostasis Model Assessment Insulin Resistance (HOMA-IR) [153,154].

Additionally, cardioprotective effects have been attributed to resveratrol. In this sense, it is suggested that resveratrol improves the endothelial function by producing nitric oxide (NO) through increasing the activity and expression of eNOS. This effect is thought to be conducted through activation of nicotinamide adenine dinucleotide-dependent deacetylase sirtuin-1 (Sirt 1) and 5′ AMP-activated protein kinase (AMPK) [155]. Besides, resveratrol exerts endothelial protection by stimulation of NF-E2-related factor 2 (Nrf2) [156] and decreasing the expression of adhesion proteins such as ICAM-1 and VCAM-1 [152].

Finally, it has been described that resveratrol may have a role in preventing obesity as it has been related with energy metabolism improvement, increasing lipolysis and reducing lipogenesis [157]. However, more studies are needed in order to corroborate these findings.

### 3.5. Tocopherol

Tocopherols, also known as vitamin E, are a family of eight fat-soluble phenolic compounds whose main dietary sources are vegetable oils, nuts and seeds [130,158].

For a long time, it has been suggested that vitamin E could prevent different metabolic diseases as a potent antioxidant, acting as scavenger of lipid peroxyl radicals by hydrogen donating [159]. In this sense, it was described that tocopherols inhibit peroxidation of membrane phospholipids and prevent generation of free radicals in cell membranes [160].

Moreover, it has been shown that supplementation with α-tocopherol or γ-tocopherol, two of the different isoforms of vitamin E, could have an effect on inflammation status by reducing CRP levels [161]. Additionally, inhibition of COX and protein kinase C (PKC) and reduction of cytokines such as IL-8 or plasminogen activator inhibitor-1 (PAI-1) are other mechanisms that may contribute to these anti-inflammatory effects [162,163].

However, the beneficial effects attributed to this vitamin previously have lately became controversial as different clinical trials have not come across such benefits, but ineffective or even harmful effects have been observed [164]. It has been recently suggested that this may be explained by the fact that vitamin E may lose most of the antioxidant capacity when ingested by human beings through different mechanisms [162].

### 3.6. Anthocyanins

Anthocyanins are water-soluble polyphenolic compounds responsible for the red, blue and purple colors of berries, black currants, black grapes, peaches, cherries, plums, pomegranate, eggplant, black beans, red radishes, red onions, red cabbage, purple corn or purple sweet potatoes [165,166,167]. Actually, they are the most abundant polyphenols in fruits and vegetables [167]. Moreover, they can also be found in teas, honey, nuts, olive oil, cocoa, and cereals [168].

These compounds have high antioxidant capacity inhibiting or decreasing free radicals by donating or transferring electrons from hydrogen atoms [167].

Regarding clinical studies, it has been shown that these bioactive compounds may prevent type 2 diabetes development by improving insulin sensitivity [169]. The exact mechanisms by which anthocyanins exert their antidiabetic effect are not yet clear, but an enhancement of the glucose uptake by muscle and adipocyte cells in an insulin-independent manner has been suggested [169].

Moreover, it has been shown that anthocyanins may have the capacity to prevent CVD development by improving endothelial function via increasing brachial artery flow-mediated dilation and HDL-c, and decreasing serum VCAM-1 and LDL-c concentrations [170,171,172,173].

Finally, these polyphenolic compounds may exert anti-inflamatory effects via reducing proinflamatory molecules such as IL-8, IL-1β or CRP [172,174].

However, most studies have used anthocyanin-rich extracts instead of purified anthocyanins; thus, a synergic effect with other polyphenols cannot be discarded.

### 3.7. Catechins

Catechins are polyphenols that can be found in a variety of foods including fruits, vegetables, chocolate, wine, and tea [175]. The epigallocatechin 3-gallate present in tea leaves is the catechin class most studied [176].

Anti-obesity effects have been attributed to these polyphenols in different studies [176]. The mechanisms of action suggested to explain these beneficial effects on body weight are: increasing energy expenditure and fat oxidation, and reducing fat absorption [177]. It is thought that energy expenditure is enhanced by catechol-*O*-methyltransferase and phosphodiesterase inhibition, which stimulates the sympathetic nervous system causing an activation of the brown adipose tissue [178]. Fat oxidation is mediated by upregulation of acyl-CoA dehydrogenase and peroxisomal b-oxidation enzymes [178,179].

Moreover, catechin intake has also been associated with lower risk of CVD development by improving lipid biomarkers. Thus, it has been reported that consumption of this kind of polyphenols may increase HDL-c and decrease LDL-c and total cholesterol [180].

Finally, and antidiabetic effect has also been related to catechin comsumption, lowering fasting glucose levels [175] and improving insulin sensitivity [178].

## 4. Conclusions

**Figure 1:**
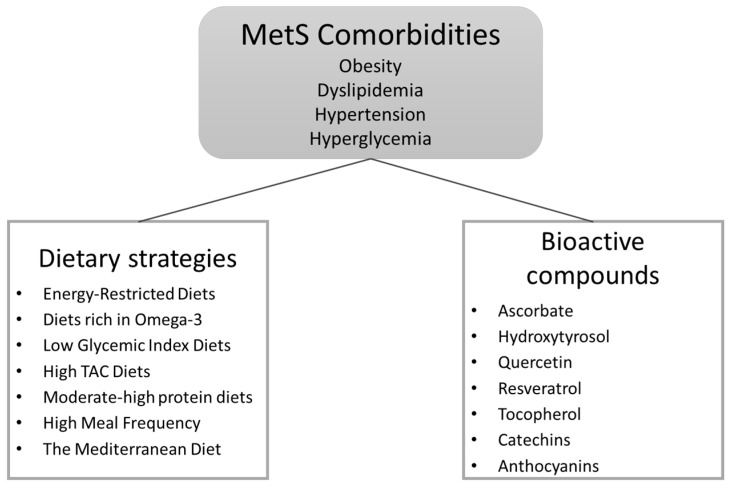
Diagram of Metabolic syndrome comorbidities and dietary strategies and bioactive compounds described.

As the prevalence of MetS reaches epidemic rates, the finding of an effective and easy-to-follow dietary strategy to combat this heterogenic disease is still a pending subject. This work recompiled different dietary nutrients and nutritional patterns with potential benefits in the prevention and treatment of MetS and related comorbidities (Figure 1) with the aim of facilitating future clinical studies in this area. The challenge now is to introduce precision bioactive compounds in personalized nutritional patterns in order to gain the most benefits for prevention and treatment of this disease through nutrition.

## Figures and Tables

**Table 1:** Criteria to define metabolic syndrome (MetS) depending on different organisms.

World Health Organization (1994) [2]
**One of these:** - Type 2 diabetes, insulin resistance or impaired glucose tolerance. **Plus at least two of the following:** - TG ≥ 1.7 mmol/L and/or HDL-c < 0.9 mmol/L (men) and < 1.0 mmol/L (women). - Urine albumin excretion > 20 µg/min or albumin:creatinine ratio > 30 mg/g. - SBP ≥ 140 mmHg or DBP ≥ 90 mmHg or treatment for hypertension. - Central obesity: BMI ≥ 30 kg/m^2^ or waist:hip ratio > 0.90 (men), > 0.85 (women).
**European Group of Insulin Resistance (1999) [3]**
- Insulin resistance defined as the top 25% of the fasting insulin values among nondiabetic individuals. **Plus at least two of the following:** - Central obesity: waist circumference ≥ 94 cm (men), ≥ 80 (women). - TG ≥ 2.0 mmol/L and/or HDL-c < 1.0 mmol/L or specific treatment. - SBP ≥ 140 mmHg or DBP ≥ 90 mmHg or treatment for hypertension. - Fasting glucose ≥ 6.1 mmol/L.
**National Cholesterol Education Program Adult Treatment Panel III (2001) [4]**
**At least three of the following:** - Abdominal obesity: waist circumference > 102 cm (men), > 88 cm (women). - TG ≥ 1.7 mmol/L. - HDL-c < 1.03 mmol/L in men, < 1.3 mmol/L in women. - SBP ≥ 130 mmHg or DBP ≥ 85 mmHg. - Fasting plasma glucose ≥ 6.1 mmol/L.
**American Heart Association/National Heart, Lung, and Blood Institute (2005) [5]**
**At least three of the following:** - Waist circumference ≥ 102 cm (men), ≥ 88 cm (women) or diagnosed type 2 diabetes. - TG ≥ 1.7 mmol/L or specific treatment for hypertriglyceridemia. - HDL-c < 1.03 mmol/L in men, < 1.3 mmol/L in women or specific treatment. - SBP ≥ 130 mmHg or DBP ≥ 85 mmHg or drug treatment for hypertension. - Fasting plasma glucose ≥ 5.6 mmol/L.
**International Diabetes Federation (2005) [6]**
- Central obesity: waist circumference ≥ 94 cm (Europids men), ≥ 80 (Europids women). **Plus at least two of the following:** - TG ≥ 1.7 mmol/L or specific treatment for hypertriglyceridemia. - HDL-c < 1.03 mmol/L in men, < 1.3 mmol/L in women or specific treatment. - SBP ≥ 130 mmHg or DBP ≥ 85 mmHg or drug treatment for hypertension. - Fasting plasma glucose ≥ 5.6 mmol/L or type 2 diabetes previously diagnosed.

BMI, body mass index; DBP, diastolic blood pressure; HDL-c, high density lipoprotein-cholesterol; SBP, systolic blood pressure; TG, triglycerides.

**Table 2:** Potential beneficial effects of different dietary patterns on the treatment of MetS comorbidities.

Dietary Pattern	Metabolic Diseases Improved	Mechanisms Implicated	Ref.
Energy-Restricted Diets	Obesity	Lipolysis	[32,33,34,35,36,37]
	Type 2 diabetes	Improvements of glycaemia and insulin resistance	
	Inflammation	↓ Inflammatory markers (e.g., IL-6)	
	CV diseases	Improvement of cholesterol profile and ↓ SBP, DBP and TG	
Diets Rich in Omega-3	Inflammation	↓ Pro-inflammatory cytokines (e.g., IL-6, TNFα)	[40,42,43,44]
	CV diseases	↓ TG, sdLDL particles	
Low Glycemic Index Diets	Type 2 diabetes	↓ HbA1c and fructosamine	[46,52,53]
High TAC Diets	Oxidative stress	Free radicals’ scavenger	[58,59,60]
Moderate-High Protein Diets	Obesity	↑ Satiety and thermogenesis	[73,75,78,79,86]
High Meal Frequency	Obesity	↓ Plasma glucose levels oscillations and ↓ insulin secretion	[93,99,100]
The Mediterranean Diet	Type 2 diabetes	Glycemic control, ↓ HbA1c, ↓ fasting glucose levels	[107]
	CV diseases	↓ TC, LDL-c, TG, and ↑ HDL-c	[111,112,113,114,115]
	Obesity	↑ satiety and ↓ body weight and waist circumference	[108,116,117]

BP, blood pressure; CV, cardiovascular; DBP, systolic blood pressure; HbA1c, glycated hemoglobin; HDL-c, high density lipoprotein cholesterol; IL-6, interleukin-6; LDL-c, low density lipoprotein cholesterol; SBP, diastolic blood pressure; sdLDL particles, small dense low density lipoprotein particles; TAC, total antioxidant capacity; TC, total cholesterol; TG, triglycerides; TNFα, tumor necrosis factor-alpha; ↓, reduction; ↑, increment.

**Table 3:** Dietary bioactive compounds with potential positive effects on MetS, biological effects and molecular mechanisms of action involved.

Bioactive Component	Metabolite Class	Biological Effects	Mechanisms	Ref.
Anthocyanins	Polyphenol	Antidiabetic	↑ Glucose uptake in an insulin-independent mechanism	[169]
		Cardioprotective	↑ BAFMD, HDL-c and ↓ VCAM-1, LDL-c	[170,171,172,173]
		Antiinflamatory	↓ IL-8, IL-1β or CRP	[172,174]
Ascorbate	Vitamin	Antioxidant	Scavenger of free radicals and regeneration of oxidized molecules	[122,124,125,127,128]
		Anti-inflammatory	↓ CRP	
		Cardioprotective	↑ eNOS and ↓ HDL-c glycation	
		Antidiabetic	↑ SVCTs	
Catechin	Polyphenol	Anti-obesity	↑ ACAD and peroxisomal β-oxidation enzymes, ↓ COMT and PDE	[177,178,180]
		Cardioprotective	↑ HDL-c and ↓ LDL-c, TC	
		Antidiabetic	↓ Fasting glucose levels and insulin sensitivity improvement	
Hydroxytyrosol	Polyphenol	Antioxidant	Free radical scavenger, radical chain breaker, and metal chelator	[132,133,134,135,136,137,138,140]
		Anti-inflammatory	↑ eNOS, ↓ COX	
		Cardioprotective	↑ HDL-c, ↓ LDL-c oxidation, ICAM-1, VCAM-1, LDL-c and TC	
Quercetin	Polyphenol	Antioxidant	↓ lipid peroxidation, ↑ antioxidant enzymes (e.g., SOD, CAT, GPX)	[142,143,144,145,146,148]
		Anti-inflammatory	↓ PI3K, GLUT2, NFκB, TNF-α, MAPK, IL-6, IL-1β, IL-8 or MCP-1	
		Anti-obesity	↓ Adipogenesis through ↑ AMPK and ↓ C/EBPα, PPARγ, and SREBP-1	
		Antidiabetic	PPARγ, GLUT2, PI3K and TK	
		↓ Blood pressure	↑ eNOS and ↓ platelet aggregation	
Resveratrol	Polyphenol	Antioxidant	Scavenger of hydroxyl, superoxide, and metal-induced radicals	[150,151,152,155,156,157]
		Anti-inflammatory	↓ NFκB, IL6, IL8, TNF-α, MCP-1, eNOS, COX	
		Cardioprotective	↑ NO and Nrf2, ↓ ICAM-1, VCAM-1	
		Anti-obesity	↑ Lipolysis, ↓ lipogenesis	
		Anti-inflammatory	↓ CRP, COX, PKC, IL-8, PAI-1	
		Antiatherogenic	↓ oxidation of LDL-c and PUFAs	
Tocopherol	Vitamin	Antioxidant	↓ lipid peroxyl radicals	[159,160,161,162,163]
		Anti-inflammatory	↓ CRP, COX, PKC, IL-8	

ACAD, acyl-CoA dehydrogenase; AFMD, artery flow-mediated dilation; AMPK, AMP-activated protein kinase; BAFMD, brachial artery flow-mediated dilation; CAT, catalase; C/EBPα, CCAAT-enhancer-binding protein-α; COMT, catechol-*O*-methyltransferase; COX, cyclooxygenase; CRP, C reactive protein; eNOS; endothelial nitric oxide synthase; GLUT2, glucose transporter 2; GPX, glutathione peroxidase; HDL-c, high density lipoprotein-cholesterol; ICAM-1, intercellular adhesion molecule; IL, interleukin; LDL-c, low density lipoprotein-cholesterol; MAPK, mitogen-activated protein kinases; MCP-1, monocyte chemoattractant protein-1; NFκB, nuclear factor kappa-light-chain-enhancer of activated B cells; NO; nitric oxide; Nrf2, NF-E2-related factor 2; PAI-1, activator inhibitor-1; PDE, phosphodiesterase; PI3K, phosphatidylinositol-3-kinase; PKC, protein kinase C; PPARγ, peroxisome proliferator-activated receptor gamma; PUFAs, polyunsaturated fatty acids; SOD, superoxide dismutase; SREBP-1; sterol regulatory element-binding protein 1; SVCTs, sodium-dependent vitamin C transporters; TC, total cholesterol; TK, tyrosine kinase; TNF-α, tumor necrosis factor α; VCAM-1, vascular cell adhesion protein 1; ↓, reduction; ↑, increment.

## References

[1] Sarafidis P.A., Nilsson P.M. (2006). The metabolic syndrome: A glance at its history. J. Hypertens..

[2] Alberti K.G., Zimmet P.Z. (1998). Definition, diagnosis and classification of diabetes mellitus and its complications. Part 1: Diagnosis and classification of diabetes mellitus provisional report of a WHO consultation. Diabet. Med..

[3] Balkau B., Charles M.A. (1999). Comment on the provisional report from the WHO consultation. European Group for the Study of Insulin Resistance (EGIR). Diabet. Med..

[4] Expert Panel on Detection, Evaluation, and Treatment of High Blood Cholesterol in Adults (2001). Executive Summary of The Third Report of The National Cholesterol Education Program (NCEP) Expert Panel on Detection, Evaluation, and Treatment of High Blood Cholesterol in Adults (Adult Treatment Panel III). JAMA.

[5] Grundy S.M., Cleeman J.I., Daniels S.R., Donato K.A., Eckel R.H., Franklin B.A., Gordon D.J., Krauss R.M., Savage P.J., Smith S.C. (2005). Diagnosis and management of the metabolic syndrome: An American Heart Association/National Heart, Lung, and Blood Institute Scientific Statement. Circulation.

[6] Alberti K.G., Zimmet P., Shaw J. (2005). The metabolic syndrome—A new worldwide definition. Lancet.

[7] Selassie M., Sinha A.C. (2011). The epidemiology and aetiology of obesity: A global challenge. Best Pract. Res. Clin. Anaesthesiol..

[8] WHO, W.H.O.. http://www.who.int/mediacentre/factsheets/fs311/es/.

[9] Shimano H. (2012). Novel qualitative aspects of tissue fatty acids related to metabolic regulation: Lessons from Elovl6 knockout. Prog. Lipid Res..

[10] Bosomworth N.J. (2013). Approach to identifying and managing atherogenic dyslipidemia: A metabolic consequence of obesity and diabetes. Can. Fam. Phys..

[11] Vidal-Puig A., Oresic M. (2014). The Metabolic Syndrome and its Complex Pathophysiology. A Systems Biology Approach to Study Metabolic Syndrome.

[12] Poitout V., Robertson R.P. (2008). Glucolipotoxicity: Fuel excess and beta-cell dysfunction. Endocr. Rev..

[13] Rizza W., Veronese N., Fontana L. (2014). What are the roles of calorie restriction and diet quality in promoting healthy longevity?. Ageing Res. Rev..

[14] Lloyd-Jones D.M., Levy D., Black H.R., Elliott W.J. (2013). Epidemiology of Hypertension. Hypertension: A Companion to Braunwald’s Heart Disease.

[15] Zanchetti A. (2014). Challenges in hypertension: Prevalence, definition, mechanisms and management. J. Hypertens..

[16] Thomas G., Shishehbor M., Brill D., Nally J.V. (2014). New hypertension guidelines: One size fits most?. Clevel. Clin. J. Med..

[17] James P.A., Oparil S., Carter B.L., Cushman W.C., Dennison-Himmelfarb C., Handler J., Lackland D.T., LeFevre M.L., MacKenzie T.D., Ogedegbe O. (2014). 2014 evidence-based guideline for the management of high blood pressure in adults: Report from the panel members appointed to the Eighth Joint National Committee (JNC 8). JAMA.

[18] Klandorf H., Chirra A.R., DeGruccio A., Girman D.J. (1989). Dimethyl sulfoxide modulation of diabetes onset in NOD mice. Diabetes.

[19] Ballard K.D., Mah E., Guo Y., Pei R., Volek J.S., Bruno R.S. (2013). Low-fat milk ingestion prevents postprandial hyperglycemia-mediated impairments in vascular endothelial function in obese individuals with metabolic syndrome. J. Nutr..

[20] Pugliese G., Solini A., Bonora E., Orsi E., Zerbini G., Fondelli C., Gruden G., Cavalot F., Lamacchia O., Trevisan R. (2014). Distribution of cardiovascular disease and retinopathy in patients with type 2 diabetes according to different classification systems for chronic kidney disease: A cross-sectional analysis of the renal insufficiency and cardiovascular events (RIACE) Italian multicenter study. Cardiovasc. Diabetol..

[21] Asif M. (2014). The prevention and control the type-2 diabetes by changing lifestyle and dietary pattern. J. Educ. Health Promot..

[22] Russell W.R., Baka A., Bjorck I., Delzenne N., Gao D., Griffiths H.R., Hadjilucas E., Juvonen K., Lahtinen S., Lansink M. (2016). Impact of Diet Composition on Blood Glucose Regulation. Crit. Rev. Food Sci. Nutr..

[23] Soares R., Costa C. (2009). Oxidative Stress, Inflammation and Angiogenesis in the Metabolic Syndrome.

[24] Rahal A., Kumar A., Singh V., Yadav B., Tiwari R., Chakraborty S., Dhama K. (2014). Oxidative Stress, Prooxidants, and Antioxidants: The Interplay. BioMed Res. Int..

[25] Parthasarathy S., Litvinov D., Selvarajan K., Garelnabi M. (2008). Lipid peroxidation and decomposition—Conflicting roles in plaque vulnerability and stability. Biochim. Biophys. Acta.

[26] McGrowder D., Riley C., Morrison E.Y., Gordon L. (2011). The role of high-density lipoproteins in reducing the risk of vascular diseases, neurogenerative disorders, and cancer. Cholesterol.

[27] Ferri N., Ruscica M. (2016). Proprotein convertase subtilisin/kexin type 9 (PCSK9) and metabolic syndrome: Insights on insulin resistance, inflammation, and atherogenic dyslipidemia. Endocrine.

[28] Oresic M., Vidal-Puig A. (2014). A Systems Biology Approach to Study Metabolic Syndrome.

[29] Lee E.G., Choi J.H., Kim K.E., Kim J.H. (2014). Effects of a Walking Program on Self-management and Risk Factors of Metabolic Syndrome in Older Korean Adults. J. Phys. Ther. Sci..

[30] Bernabe G.J., Zafrilla R.P., Mulero C.J., Gomez J.P., Leal H.M., Abellan A.J. (2013). Biochemical and nutritional markers and antioxidant activity in metabolic syndrome. Endocrinol. Nutr..

[31] Bales C.W., Kraus W.E. (2013). Caloric restriction: Implications for human cardiometabolic health. J. Cardiopulm. Rehabil. Prev..

[32] Grams J., Garvey W.T. (2015). Weight Loss and the Prevention and Treatment of Type 2 Diabetes Using Lifestyle Therapy, Pharmacotherapy, and Bariatric Surgery: Mechanisms of Action. Curr. Obes. Rep..

[33] Lazo M., Solga S.F., Horska A., Bonekamp S., Diehl A.M., Brancati F.L., Wagenknecht L.E., Pi-Sunyer F.X., Kahn S.E., Clark J.M. (2010). Effect of a 12-month intensive lifestyle intervention on hepatic steatosis in adults with type 2 diabetes. Diabetes Care.

[34] Rossmeislova L., Malisova L., Kracmerova J., Stich V. (2013). Adaptation of human adipose tissue to hypocaloric diet. Int. J. Obes..

[35] Wing R.R., Lang W., Wadden T.A., Safford M., Knowler W.C., Bertoni A.G., Hill J.O., Brancati F.L., Peters A., Wagenknecht L. (2011). Benefits of modest weight loss in improving cardiovascular risk factors in overweight and obese individuals with type 2 diabetes. Diabetes Care.

[36] Golay A., Brock E., Gabriel R., Konrad T., Lalic N., Laville M., Mingrone G., Petrie J., Phan T.M., Pietilainen K.H. (2013). Taking small steps towards targets—Perspectives for clinical practice in diabetes, cardiometabolic disorders and beyond. Int. J. Clin. Pract..

[37] Fock K.M., Khoo J. (2013). Diet and exercise in management of obesity and overweight. J. Gastroenterol. Hepatol..

[38] Abete I., Parra D., Martinez J.A. (2008). Energy-restricted diets based on a distinct food selection affecting the glycemic index induce different weight loss and oxidative response. Clin. Nutr..

[39] Alberti K.G., Eckel R.H., Grundy S.M., Zimmet P.Z., Cleeman J.I., Donato K.A., Fruchart J.C., James W.P., Loria C.M., Smith S.C. (2009). Harmonizing the metabolic syndrome: A joint interim statement of the International Diabetes Federation Task Force on Epidemiology and Prevention; National Heart, Lung, and Blood Institute; American Heart Association; World Heart Federation; International Atherosclerosis Society; and International Association for the Study of Obesity. Circulation.

[40] Fleming J.A., Kris-Etherton P.M. (2014). The evidence for alpha-linolenic acid and cardiovascular disease benefits: Comparisons with eicosapentaenoic acid and docosahexaenoic acid. Adv. Nutr..

[41] Gray B., Steyn F., Davies P.S., Vitetta L. (2013). Omega-3 fatty acids: A review of the effects on adiponectin and leptin and potential implications for obesity management. Eur. J. Clin. Nutr..

[42] Wen Y.T., Dai J.H., Gao Q. (2014). Effects of Omega-3 fatty acid on major cardiovascular events and mortality in patients with coronary heart disease: A meta-analysis of randomized controlled trials. Nutr. Metab. Cardiovasc. Dis..

[43] Lopez-Huertas E. (2012). The effect of EPA and DHA on metabolic syndrome patients: A systematic review of randomised controlled trials. Br. J. Nutr..

[44] Maiorino M.I., Chiodini P., Bellastella G., Giugliano D., Esposito K. (2016). Sexual dysfunction in women with cancer: A systematic review with meta-analysis of studies using the Female Sexual Function Index. Endocrine.

[45] EFSA NDA Panel (EFSA Panel on Dietetic Products, Nutrition and Allergies) (2010). Scientific Opinion on Dietary Reference Values for fats, including saturated fatty acids, polyunsaturated fatty acids, monounsaturated fatty acids, trans fatty acids, and cholesterol1. EFSA J..

[46] Bellastella G., Bizzarro A., Aitella E., Barrasso M., Cozzolino D., di Martino S., Esposito K., de Bellis A. (2015). Pregnancy may favour the development of severe autoimmune central diabetes insipidus in women with vasopressin cell antibodies: Description of two cases. Eur. J. Endocrinol..

[47] Sun F.H., Li C., Zhang Y.J., Wong S.H., Wang L. (2016). Effect of Glycemic Index of Breakfast on Energy Intake at Subsequent Meal among Healthy People: A Meta-Analysis. Nutrients.

[48] Barclay A.W., Brand-Miller J.C., Wolever T.M. (2005). Glycemic index, glycemic load, and glycemic response are not the same. Diabetes Care.

[49] Nakagawa T., Hu H., Zharikov S., Tuttle K.R., Short R.A., Glushakova O., Ouyang X., Feig D.I., Block E.R., Herrera-Acosta J. (2006). A causal role for uric acid in fructose-induced metabolic syndrome. Am. J. Physiol. Ren. Physiol..

[50] Symons Downs D., Hausenblas H.A. (2004). Women’s exercise beliefs and behaviors during their pregnancy and postpartum. J. Midwifery Women Health.

[51] Brand-Miller J., McMillan-Price J., Steinbeck K., Caterson I. (2009). Dietary glycemic index: Health implications. J. Am. Coll. Nutr..

[52] Thomas D., Elliott E.J. (2009). Low glycaemic index, or low glycaemic load, diets for diabetes mellitus. Cochrane Database Syst. Rev..

[53] Barrea L., Balato N., di Somma C., Macchia P.E., Napolitano M., Savanelli M.C., Esposito K., Colao A., Savastano S. (2015). Nutrition and psoriasis: Is there any association between the severity of the disease and adherence to the Mediterranean diet?. J. Transl. Med..

[54] Mathias K.C., Ng S.W., Popkin B. (2015). Monitoring changes in the nutritional content of ready-to-eat grain-based dessert products manufactured and purchased between 2005 and 2012. J. Acad. Nutr. Diet..

[55] Serafini M., del Rio D. (2004). Understanding the association between dietary antioxidants, redox status and disease: Is the Total Antioxidant Capacity the right tool?. Redox Rep..

[56] Bellastella G., Maiorino M.I., Olita L., della Volpe E., Giugliano D., Esposito K. (2015). Premature ejaculation is associated with glycemic control in Type 1 diabetes. J. Sex. Med..

[57] Zulet M.A., Moreno-Aliaga M.J., Martinez J.A., Symonds M.E. (2012). Dietary Determinants of Fat Mass and Body Composition. Adipose Tissue Biology.

[58] Carlsen M.H., Halvorsen B.L., Holte K., Bohn S.K., Dragland S., Sampson L., Willey C., Senoo H., Umezono Y., Sanada C. (2010). The total antioxidant content of more than 3100 foods, beverages, spices, herbs and supplements used worldwide. Nutr. J..

[59] Harasym J., Oledzki R. (2014). Effect of fruit and vegetable antioxidants on total antioxidant capacity of blood plasma. Nutrition.

[60] Maiorino M.I., Bellastella G., Petrizzo M., della Volpe E., Orlando R., Giugliano D., Esposito K. (2015). Circulating endothelial progenitor cells in type 1 diabetic patients with erectile dysfunction. Endocrine.

[61] Bahadoran Z., Golzarand M., Mirmiran P., Shiva N., Azizi F. (2012). Dietary total antioxidant capacity and the occurrence of metabolic syndrome and its components after a 3-year follow-up in adults: Tehran Lipid and Glucose Study. Nutr. Metab..

[62] Chrysohoou C., Esposito K., Giugliano D., Panagiotakos D.B. (2015). Peripheral Arterial Disease and Cardiovascular Risk: The Role of Mediterranean Diet. Angiology.

[63] De la Iglesia R., Lopez-Legarrea P., Celada P., Sanchez-Muniz F.J., Martinez J.A., Zulet M.A. (2013). Beneficial effects of the RESMENA dietary pattern on oxidative stress in patients suffering from metabolic syndrome with hyperglycemia are associated to dietary TAC and fruit consumption. Int. J. Mol. Sci..

[64] Lopez-Legarrea P., de la Iglesia R., Abete I., Bondia-Pons I., Navas-Carretero S., Forga L., Martinez J.A., Zulet M.A. (2013). Short-term role of the dietary total antioxidant capacity in two hypocaloric regimes on obese with metabolic syndrome symptoms: The RESMENA randomized controlled trial. Nutr. Metab..

[65] Puchau B., Zulet M.A., de Echavarri A.G., Hermsdorff H.H., Martinez J.A. (2010). Dietary total antioxidant capacity is negatively associated with some metabolic syndrome features in healthy young adults. Nutrition.

[66] World Health Organization (2000). Obesity: Preventing and Managing the Global Epidemic.

[67] Tapsell L.C., Hemphill I., Cobiac L., Patch C.S., Sullivan D.R., Fenech M., Roodenrys S., Keogh J.B., Clifton P.M., Williams P.G. (2006). Health benefits of herbs and spices: The past, the present, the future. Med. J. Aust..

[68] Abete I., Astrup A., Martinez J.A., Thorsdottir I., Zulet M.A. (2010). Obesity and the metabolic syndrome: Role of different dietary macronutrient distribution patterns and specific nutritional components on weight loss and maintenance. Nutr. Rev..

[69] Ebbeling C.B., Swain J.F., Feldman H.A., Wong W.W., Hachey D.L., Garcia-Lago E., Ludwig D.S. (2012). Effects of dietary composition on energy expenditure during weight-loss maintenance. JAMA.

[70] Abete I., Goyenechea E., Zulet M.A., Martinez J.A. (2011). Obesity and metabolic syndrome: Potential benefit from specific nutritional components. Nutr. Metab. Cardiovasc. Dis..

[71] Arciero P.J., Ormsbee M.J., Gentile C.L., Nindl B.C., Brestoff J.R., Ruby M. (2013). Increased protein intake and meal frequency reduces abdominal fat during energy balance and energy deficit. Obesity.

[72] Wikarek T., Chudek J., Owczarek A., Olszanecka-Glinianowicz M. (2014). Effect of dietary macronutrients on postprandial incretin hormone release and satiety in obese and normal-weight women. Br. J. Nutr..

[73] Bray G.A., Smith S.R., de Jonge L., Xie H., Rood J., Martin C.K., Most M., Brock C., Mancuso S., Redman L.M. (2012). Effect of dietary protein content on weight gain, energy expenditure, and body composition during overeating: A randomized controlled trial. JAMA.

[74] Westerterp-Plantenga M.S., Nieuwenhuizen A., Tome D., Soenen S., Westerterp K.R. (2009). Dietary protein, weight loss, and weight maintenance. Annu. Rev. Nutr..

[75] Koppes L.L., Boon N., Nooyens A.C., van Mechelen W., Saris W.H. (2009). Macronutrient distribution over a period of 23 years in relation to energy intake and body fatness. Br. J. Nutr..

[76] De Jonge L., Bray G.A., Smith S.R., Ryan D.H., de Souza R.J., Loria C.M., Champagne C.M., Williamson D.A., Sacks F.M. (2012). Effect of diet composition and weight loss on resting energy expenditure in the POUNDS LOST study. Obesity.

[77] Stocks T., Angquist L., Hager J., Charon C., Holst C., Martinez J.A., Saris W.H., Astrup A., Sorensen T.I., Larsen L.H. (2013). TFAP2B-dietary protein and glycemic index interactions and weight maintenance after weight loss in the DiOGenes trial. Hum. Hered..

[78] Giugliano D., Maiorino M.I., Esposito K. (2015). Linking prediabetes and cancer: A complex issue. Diabetologia.

[79] Bendtsen L.Q., Lorenzen J.K., Bendsen N.T., Rasmussen C., Astrup A. (2013). Effect of dairy proteins on appetite, energy expenditure, body weight, and composition: A review of the evidence from controlled clinical trials. Adv. Nutr..

[80] Heer M., Egert S. (2015). Nutrients other than carbohydrates: Their effects on glucose homeostasis in humans. Diabetes Metab. Res. Rev..

[81] Layman D.K., Evans E.M., Erickson D., Seyler J., Weber J., Bagshaw D., Griel A., Psota T., Kris-Etherton P. (2009). A moderate-protein diet produces sustained weight loss and long-term changes in body composition and blood lipids in obese adults. J. Nutr..

[82] Pedersen A.N., Kondrup J., Borsheim E. (2013). Health effects of protein intake in healthy adults: A systematic literature review. Food Nutr. Res..

[83] Daly R.M., O’Connell S.L., Mundell N.L., Grimes C.A., Dunstan D.W., Nowson C.A. (2014). Protein-enriched diet, with the use of lean red meat, combined with progressive resistance training enhances lean tissue mass and muscle strength and reduces circulating IL-6 concentrations in elderly women: A cluster randomized controlled trial. Am. J. Clin. Nutr..

[84] Arciero P.J., Gentile C.L., Pressman R., Everett M., Ormsbee M.J., Martin J., Santamore J., Gorman L., Fehling P.C., Vukovich M.D. (2008). Moderate protein intake improves total and regional body composition and insulin sensitivity in overweight adults. Metab. Clin. Exp..

[85] Gregory S.M., Headley S.A., Wood R.J. (2011). Effects of dietary macronutrient distribution on vascular integrity in obesity and metabolic syndrome. Nutr. Rev..

[86] Consenso FESNAD-SEEDO (2011). Recomendaciones nutricionales basadas en la evidencia para la prevención y el tratamiento del sobrepeso y la obesidad en adultos (Consenso FESNAD-SEEDO). Rev. Esp. Obes..

[87] Jakubowicz D., Froy O., Wainstein J., Boaz M. (2012). Meal timing and composition influence ghrelin levels, appetite scores and weight loss maintenance in overweight and obese adults. Steroids.

[88] Schwarz N.A., Rigby B.R., La Bounty P., Shelmadine B., Bowden R.G. (2011). A review of weight control strategies and their effects on the regulation of hormonal balance. J. Nutr. Metab..

[89] Ohkawara K., Cornier M.A., Kohrt W.M., Melanson E.L. (2013). Effects of increased meal frequency on fat oxidation and perceived hunger. Obesity.

[90] Ekmekcioglu C., Touitou Y. (2011). Chronobiological aspects of food intake and metabolism and their relevance on energy balance and weight regulation. Obes. Rev..

[91] Lioret S., Touvier M., Lafay L., Volatier J.L., Maire B. (2008). Are eating occasions and their energy content related to child overweight and socioeconomic status?. Obesity.

[92] Bhutani S., Varady K.A. (2009). Nibbling versus feasting: Which meal pattern is better for heart disease prevention?. Nutr. Rev..

[93] Leidy H.J., Tang M., Armstrong C.L., Martin C.B., Campbell W.W. (2011). The effects of consuming frequent, higher protein meals on appetite and satiety during weight loss in overweight/obese men. Obesity.

[94] Mills J.P., Perry C.D., Reicks M. (2011). Eating frequency is associated with energy intake but not obesity in midlife women. Obesity.

[95] Cameron J.D., Cyr M.J., Doucet E. (2010). Increased meal frequency does not promote greater weight loss in subjects who were prescribed an 8-week equi-energetic energy-restricted diet. Br. J. Nutr..

[96] Smeets A.J., Lejeune M.P., Westerterp-Plantenga M.S. (2009). Effects of oral fat perception by modified sham feeding on energy expenditure, hormones and appetite profile in the postprandial state. Br. J. Nutr..

[97] Taylor M.A., Garrow J.S. (2001). Compared with nibbling, neither gorging nor a morning fast affect short-term energy balance in obese patients in a chamber calorimeter. Int. J. Obes. Relat. Metab. Disord..

[98] Smeets A.J., Westerterp-Plantenga M.S. (2008). Acute effects on metabolism and appetite profile of one meal difference in the lower range of meal frequency. Br. J. Nutr..

[99] Heden T.D., LeCheminant J.D., Smith J.D. (2012). Influence of weight classification on walking and jogging energy expenditure prediction in women. Res. Q. Exerc. Sport.

[100] Bachman J.L., Raynor H.A. (2012). Effects of manipulating eating frequency during a behavioral weight loss intervention: A pilot randomized controlled trial. Obesity.

[101] Perrigue M.M., Drewnowski A., Wang C.Y., Neuhouser M.L. (2016). Higher Eating Frequency Does Not Decrease Appetite in Healthy Adults. J. Nutr..

[102] Keys A. (1997). Coronary heart disease in seven countries. 1970. Nutrition.

[103] Keys A., Menotti A., Aravanis C., Blackburn H., Djordevic B.S., Buzina R., Dontas A.S., Fidanza F., Karvonen M.J., Kimura N. (1984). The seven countries study: 2289 deaths in 15 years. Prev. Med..

[104] Davis C., Bryan J., Hodgson J., Murphy K. (2015). Definition of the Mediterranean Diet; a Literature Review. Nutrients.

[105] Sofi F., Macchi C., Abbate R., Gensini G.F., Casini A. (2014). Mediterranean diet and health status: An updated meta-analysis and a proposal for a literature-based adherence score. Public Health Nutr..

[106] Mayneris-Perxachs J., Sala-Vila A., Chisaguano M., Castellote A.I., Estruch R., Covas M.I., Fito M., Salas-Salvado J., Martinez-Gonzalez M.A., Lamuela-Raventos R. (2014). Effects of 1-year intervention with a Mediterranean diet on plasma fatty acid composition and metabolic syndrome in a population at high cardiovascular risk. PLoS ONE.

[107] Esposito K., Maiorino M.I., Bellastella G., Chiodini P., Panagiotakos D., Giugliano D. (2015). A journey into a Mediterranean diet and type 2 diabetes: A systematic review with meta-analyses. BMJ Open.

[108] Kastorini C.M., Milionis H.J., Esposito K., Giugliano D., Goudevenos J.A., Panagiotakos D.B. (2011). The effect of Mediterranean diet on metabolic syndrome and its components: A meta-analysis of 50 studies and 534,906 individuals. J. Am. Coll. Cardiol..

[109] Schwingshackl L., Missbach B., Konig J., Hoffmann G. (2015). Adherence to a Mediterranean diet and risk of diabetes: A systematic review and meta-analysis. Public Health Nutr..

[110] Koloverou E., Esposito K., Giugliano D., Panagiotakos D. (2014). The effect of Mediterranean diet on the development of type 2 diabetes mellitus: A meta-analysis of 10 prospective studies and 136,846 participants. Metab. Clin. Exp..

[111] Salas-Salvado J., Garcia-Arellano A., Estruch R., Marquez-Sandoval F., Corella D., Fiol M., Gomez-Gracia E., Vinoles E., Aros F., Herrera C. (2008). Components of the Mediterranean-type food pattern and serum inflammatory markers among patients at high risk for cardiovascular disease. Eur. J. Clin. Nutr..

[112] Martinez-Gonzalez M.A., Garcia-Lopez M., Bes-Rastrollo M., Toledo E., Martinez-Lapiscina E.H., Delgado-Rodriguez M., Vazquez Z., Benito S., Beunza J.J. (2011). Mediterranean diet and the incidence of cardiovascular disease: A Spanish cohort. Nutr. Metab. Cardiovasc. Dis..

[113] Fito M., Estruch R., Salas-Salvado J., Martinez-Gonzalez M.A., Aros F., Vila J., Corella D., Diaz O., Saez G., de la Torre R. (2014). Effect of the Mediterranean diet on heart failure biomarkers: A randomized sample from the PREDIMED trial. Eur. J. Heart Fail..

[114] Estruch R., Ros E., Salas-Salvado J., Covas M.I., Corella D., Aros F., Gomez-Gracia E., Ruiz-Gutierrez V., Fiol M., Lapetra J. (2013). Primary prevention of cardiovascular disease with a Mediterranean diet. N. Engl. J. Med..

[115] Serra-Majem L., Roman B., Estruch R. (2006). Scientific evidence of interventions using the Mediterranean diet: A systematic review. Nutr. Rev..

[116] Esposito K., Kastorini C.M., Panagiotakos D.B., Giugliano D. (2011). Mediterranean diet and weight loss: Meta-analysis of randomized controlled trials. Metab. Syndr. Relat. Disord..

[117] Razquin C., Martinez J.A., Martinez-Gonzalez M.A., Mitjavila M.T., Estruch R., Marti A. (2009). A 3 years follow-up of a Mediterranean diet rich in virgin olive oil is associated with high plasma antioxidant capacity and reduced body weight gain. Eur. J. Clin. Nutr..

[118] Bertoli S., Spadafranca A., Bes-Rastrollo M., Martinez-Gonzalez M.A., Ponissi V., Beggio V., Leone A., Battezzati A. (2015). Adherence to the Mediterranean diet is inversely related to binge eating disorder in patients seeking a weight loss program. Clin. Nutr..

[119] Rios-Hoyo A., Cortes M.J., Rios-Ontiveros H., Meaney E., Ceballos G., Gutierrez-Salmean G. (2014). Obesity, Metabolic Syndrome, and Dietary Therapeutical Approaches with a Special Focus on Nutraceuticals (Polyphenols): A Mini-Review. Int. J. Vitam. Nutr. Res..

[120] Juraschek S.P., Guallar E., Appel L.J., Miller E.R. (2012). Effects of vitamin C supplementation on blood pressure: A meta-analysis of randomized controlled trials. Am. J. Clin. Nutr..

[121] Michels A.J., Frei B. (2013). Myths, artifacts, and fatal flaws: Identifying limitations and opportunities in vitamin C research. Nutrients.

[122] Frei B., Birlouez-Aragon I., Lykkesfeldt J. (2012). Authors’ perspective: What is the optimum intake of vitamin C in humans?. Crit. Rev. Food Sci. Nutr..

[123] Mason S.A., della Gatta P.A., Snow R.J., Russell A.P., Wadley G.D. (2016). Ascorbic acid supplementation improves skeletal muscle oxidative stress and insulin sensitivity in people with type 2 diabetes: Findings of a randomized controlled study. Free Radic. Biol. Med..

[124] Chambial S., Dwivedi S., Shukla K.K., John P.J., Sharma P. (2013). Vitamin C in Disease Prevention and Cure: An Overview. Indian J. Clin. Biochem..

[125] Block G., Jensen C.D., Dalvi T.B., Norkus E.P., Hudes M., Crawford P.B., Holland N., Fung E.B., Schumacher L., Harmatz P. (2009). Vitamin C treatment reduces elevated C-reactive protein. Free Radic. Biol. Med..

[126] Ashor A.W., Siervo M., Lara J., Oggioni C., Afshar S., Mathers J.C. (2015). Effect of vitamin C and vitamin E supplementation on endothelial function: A systematic review and meta-analysis of randomised controlled trials. Br. J. Nutr..

[127] Kim S.M., Lim S.M., Yoo J.A., Woo M.J., Cho K.H. (2015). Consumption of high-dose vitamin C (1250 mg per day) enhances functional and structural properties of serum lipoprotein to improve anti-oxidant, anti-atherosclerotic, and anti-aging effects via regulation of anti-inflammatory microRNA. Food Funct..

[128] Monfared S., Larijani B., Abdollahi M. (2009). Islet transplantation and antioxidant management: A comprehensive review. World J. Gastroenterol..

[129] German Nutrition Society (DGE) (2015). New Reference Values for Vitamin C Intake. Ann. Nutr. Metab..

[130] Mamede A.C., Tavares S.D., Abrantes A.M., Trindade J., Maia J.M., Botelho M.F. (2011). The role of vitamins in cancer: A review. Nutr. Cancer.

[131] Moser M.A., Chun O.K. (2016). Vitamin C and Heart Health: A Review Based on Findings from Epidemiologic Studies. Int. J. Mol. Sci..

[132] Vilaplana-Perez C., Aunon D., Garcia-Flores L.A., Gil-Izquierdo A. (2014). Hydroxytyrosol and potential uses in cardiovascular diseases, cancer, and AIDS. Front. Nutr..

[133] Achmon Y., Fishman A. (2015). The antioxidant hydroxytyrosol: Biotechnological production challenges and opportunities. Appl. Microbiol. Biotechnol..

[134] Bulotta S., Celano M., Lepore S.M., Montalcini T., Pujia A., Russo D. (2014). Beneficial effects of the olive oil phenolic components oleuropein and hydroxytyrosol: Focus on protection against cardiovascular and metabolic diseases. J. Transl. Med..

[135] EFSA NDA Panel (EFSA Panel on Dietetic Products, Nutrition and Allergies) (2011). Scientific Opinion on the substantiation of health claims related to polyphenols in olive and protection of LDL particles from oxidative damage (ID 1333, 1638, 1639, 1696, 2865), maintenance of normal blood HDL cholesterol concentrations (ID 1639). EFSA J..

[136] Scoditti E., Nestola A., Massaro M., Calabriso N., Storelli C., De Caterina R., Carluccio M.A. (2014). Hydroxytyrosol suppresses MMP-9 and COX-2 activity and expression in activated human monocytes via PKCalpha and PKCbeta1 inhibition. Atherosclerosis.

[137] Giordano E., Dangles O., Rakotomanomana N., Baracchini S., Visioli F. (2015). 3-*O*-Hydroxytyrosol glucuronide and 4-*O*-hydroxytyrosol glucuronide reduce endoplasmic reticulum stress in vitro. Food Funct..

[138] Granados-Principal S., Quiles J.L., Ramirez-Tortosa C.L., Sanchez-Rovira P., Ramirez-Tortosa M.C. (2010). Hydroxytyrosol: From laboratory investigations to future clinical trials. Nutr. Rev..

[139] Carluccio M.A., Siculella L., Ancora M.A., Massaro M., Scoditti E., Storelli C., Visioli F., Distante A., De Caterina R. (2003). Olive oil and red wine antioxidant polyphenols inhibit endothelial activation: Antiatherogenic properties of Mediterranean diet phytochemicals. Arterioscler. Thromb. Vasc. Biol..

[140] Visioli F., Bernardini E. (2011). Extra virgin olive oil’s polyphenols: Biological activities. Curr. Pharm. Des..

[141] Nabavi S.F., Russo G.L., Daglia M., Nabavi S.M. (2015). Role of quercetin as an alternative for obesity treatment: You are what you eat!. Food Chem..

[142] Vinayagam R., Xu B. (2015). Antidiabetic properties of dietary flavonoids: A cellular mechanism review. Nutr. Metab..

[143] Shibata T., Nakashima F., Honda K., Lu Y.J., Kondo T., Ushida Y., Aizawa K., Suganuma H., Oe S., Tanaka H. (2014). Toll-like receptors as a target of food-derived anti-inflammatory compounds. J. Biol. Chem..

[144] Ahn J., Lee H., Kim S., Park J., Ha T. (2008). The anti-obesity effect of quercetin is mediated by the AMPK and MAPK signaling pathways. Biochem. Biophys. Res. Commun..

[145] Fang X.K., Gao J., Zhu D.N. (2008). Kaempferol and quercetin isolated from Euonymus alatus improve glucose uptake of 3T3-L1 cells without adipogenesis activity. Life Sci..

[146] Clark J.L., Zahradka P., Taylor C.G. (2015). Efficacy of flavonoids in the management of high blood pressure. Nutr. Rev..

[147] D’Andrea G. (2015). Quercetin: A flavonol with multifaceted therapeutic applications?. Fitoterapia.

[148] Larson A., Witman M.A., Guo Y., Ives S., Richardson R.S., Bruno R.S., Jalili T., Symons J.D. (2012). Acute, quercetin-induced reductions in blood pressure in hypertensive individuals are not secondary to lower plasma angiotensin-converting enzyme activity or endothelin-1: Nitric oxide. Nutr. Res..

[149] Tome-Carneiro J., Gonzalvez M., Larrosa M., Yanez-Gascon M.J., Garcia-Almagro F.J., Ruiz-Ros J.A., Tomas-Barberan F.A., Garcia-Conesa M.T., Espin J.C. (2013). Resveratrol in primary and secondary prevention of cardiovascular disease: A dietary and clinical perspective. Ann. N. Y. Acad. Sci..

[150] Leonard S.S., Xia C., Jiang B.H., Stinefelt B., Klandorf H., Harris G.K., Shi X. (2003). Resveratrol scavenges reactive oxygen species and effects radical-induced cellular responses. Biochem. Biophys. Res. Commun..

[151] Ren Z., Wang L., Cui J., Huoc Z., Xue J., Cui H., Mao Q., Yang R. (2013). Resveratrol inhibits NF-κB signaling through suppression of p65 and IκB kinase activities. Die Pharm..

[152] Latruffe N., Lancon A., Frazzi R., Aires V., Delmas D., Michaille J.J., Djouadi F., Bastin J., Cherkaoui-Malki M. (2015). Exploring new ways of regulation by resveratrol involving miRNAs, with emphasis on inflammation. Ann. N. Y. Acad. Sci..

[153] Hausenblas H.A., Schoulda J.A., Smoliga J.M. (2015). Resveratrol treatment as an adjunct to pharmacological management in type 2 diabetes mellitus—Systematic review and meta-analysis. Mol. Nutr. Food Res..

[154] Liu K., Zhou R., Wang B., Mi M.T. (2014). Effect of resveratrol on glucose control and insulin sensitivity: A meta-analysis of 11 randomized controlled trials. Am. J. Clin. Nutr..

[155] Bitterman J.L., Chung J.H. (2015). Metabolic effects of resveratrol: Addressing the controversies. Cell. Mol. Life Sci..

[156] Han S., Park J.S., Lee S., Jeong A.L., Oh K.S., Ka H.I., Choi H.J., Son W.C., Lee W.Y., Oh S.J. (2016). CTRP1 protects against diet-induced hyperglycemia by enhancing glycolysis and fatty acid oxidation. J. Nutr. Biochem..

[157] Gambini J., Ingles M., Olaso G., Lopez-Grueso R., Bonet-Costa V., Gimeno-Mallench L., Mas-Bargues C., Abdelaziz K.M., Gomez-Cabrera M.C., Vina J. (2015). Properties of Resveratrol: In Vitro and In Vivo Studies about Metabolism, Bioavailability, and Biological Effects in Animal Models and Humans. Oxid. Med. Cell. Longev..

[158] Yang C.S., Suh N. (2013). Cancer prevention by different forms of tocopherols. Top. Curr. Chem..

[159] Jiang Q. (2014). Natural forms of vitamin E: Metabolism, antioxidant, and anti-inflammatory activities and their role in disease prevention and therapy. Free Radic. Biol. Med..

[160] Witting P.K., Upston J.M., Stocker R., Quinn P.J., Kagan V.E. The molecular action of alpha-tocopherol in lipoprotein lipid peroxidation. Pro- and antioxidant activity of vitamin E in complex heterogeneous lipid emulsions. Fat-Soluble Vitamins.

[161] Saboori S., Shab-Bidar S., Speakman J.R., Yousefi Rad E., Djafarian K. (2015). Effect of vitamin E supplementation on serum C-reactive protein level: A meta-analysis of randomized controlled trials. Eur. J. Clin. Nutr..

[162] Azzi A., Meydani S.N., Meydani M., Zingg J.M. (2016). The rise, the fall and the renaissance of vitamin E. Arch. Biochem. Biophys..

[163] Raederstorff D., Wyss A., Calder P.C., Weber P., Eggersdorfer M. (2015). Vitamin E function and requirements in relation to PUFA. Br. J. Nutr..

[164] Loffredo L., Perri L., Di Castelnuovo A., Iacoviello L., De Gaetano G., Violi F. (2015). Supplementation with vitamin E alone is associated with reduced myocardial infarction: A meta-analysis. Nutr. Metab. Cardiovasc. Dis..

[165] Giampieri F., Tulipani S., Alvarez-Suarez J.M., Quiles J.L., Mezzetti B., Battino M. (2012). The strawberry: Composition, nutritional quality, and impact on human health. Nutrition.

[166] Amiot M.J., Riva C., Vinet A. (2016). Effects of dietary polyphenols on metabolic syndrome features in humans: A systematic review. Obes. Rev..

[167] Smeriglio A., Barreca D., Bellocco E., Trombetta D. (2016). Chemistry, Pharmacology and Health Benefits of Anthocyanins. Phytother. Res..

[168] Lila M.A. (2004). Anthocyanins and Human Health: An In Vitro Investigative Approach. J. Biomed. Biotechnol..

[169] Stull A.J., Cash K.C., Johnson W.D., Champagne C.M., Cefalu W.T. (2010). Bioactives in blueberries improve insulin sensitivity in obese, insulin-resistant men and women. J. Nutr..

[170] Zhu Y., Xia M., Yang Y., Liu F., Li Z., Hao Y., Mi M., Jin T., Ling W. (2011). Purified anthocyanin supplementation improves endothelial function via NO-cGMP activation in hypercholesterolemic individuals. Clin. Chem..

[171] Qin Y., Xia M., Ma J., Hao Y., Liu J., Mou H., Cao L., Ling W. (2009). Anthocyanin supplementation improves serum LDL- and HDL-cholesterol concentrations associated with the inhibition of cholesteryl ester transfer protein in dyslipidemic subjects. Am. J. Clin. Nutr..

[172] Zhu Y., Ling W., Guo H., Song F., Ye Q., Zou T., Li D., Zhang Y., Li G., Xiao Y. (2013). Anti-inflammatory effect of purified dietary anthocyanin in adults with hypercholesterolemia: A randomized controlled trial. Nutr. Metab. Cardiovasc. Dis..

[173] Zhu Y., Huang X., Zhang Y., Wang Y., Liu Y., Sun R., Xia M. (2014). Anthocyanin supplementation improves HDL-associated paraoxonase 1 activity and enhances cholesterol efflux capacity in subjects with hypercholesterolemia. J. Clin. Endocrinol. Metab..

[174] Karlsen A., Retterstol L., Laake P., Paur I., Bohn S.K., Sandvik L., Blomhoff R. (2007). Anthocyanins inhibit nuclear factor-kappaB activation in monocytes and reduce plasma concentrations of pro-inflammatory mediators in healthy adults. J. Nutr..

[175] Keske M.A., Ng H.L., Premilovac D., Rattigan S., Kim J.A., Munir K., Yang P., Quon M.J. (2015). Vascular and metabolic actions of the green tea polyphenol epigallocatechin gallate. Curr. Med. Chem..

[176] Johnson R., Bryant S., Huntley A.L. (2012). Green tea and green tea catechin extracts: An overview of the clinical evidence. Maturitas.

[177] Huang J., Wang Y., Xie Z., Zhou Y., Zhang Y., Wan X. (2014). The anti-obesity effects of green tea in human intervention and basic molecular studies. Eur. J. Clin. Nutr..

[178] Hursel R., Westerterp-Plantenga M.S. (2013). Catechin- and caffeine-rich teas for control of body weight in humans. Am. J. Clin. Nutr..

[179] Gutierrez-Salmean G., Ortiz-Vilchis P., Vacaseydel C.M., Rubio-Gayosso I., Meaney E., Villarreal F., Ramirez-Sanchez I., Ceballos G. (2014). Acute effects of an oral supplement of (−)-epicatechin on postprandial fat and carbohydrate metabolism in normal and overweight subjects. Food Funct..

[180] Khalesi S., Sun J., Buys N., Jamshidi A., Nikbakht-Nasrabadi E., Khosravi-Boroujeni H. (2014). Green tea catechins and blood pressure: A systematic review and meta-analysis of randomised controlled trials. Eur. J. Nutr..

